# Predictors of Extended Intensive Care Unit Utilization After Ovarian Cancer Surgery

**DOI:** 10.3390/cancers17193203

**Published:** 2025-10-01

**Authors:** Vasilis Theodoulidis, Kalliopi Kissoudi, Kimonas Chatzistamatiou, Panagiotis Tzitzis, Dimitris Zouzoulas, Iakovos Theodoulidis, Christos Anthoulakis, Freideriki Sifaki, Theodoros Moysiadis, Eleni Koraki, Grigoris Grimbizis, Dimitris Tsolakidis

**Affiliations:** 1 1st Department of Obstetrics & Gynecology, Papageorgiou General Hospital, Aristotle University of Thessaloniki, 564 29 Thessaloniki, Greece; 2 Anesthesiology Department, Papageorgiou General Hospital, 564 29 Thessaloniki, Greece; 3 Department of Computer Science, School of Sciences and Engineering, University of Nicosia, 2417 Nicosia, Cyprus

**Keywords:** ovarian cancer surgery, intensive care unit, prolonged ICU stay, predictors

## Abstract

**Background:** Patients with ovarian cancer often require intensive care unit (ICU) admission after surgery due to the complexity of the operation and the higher risk of complications. However, not all patients require a prolonged stay in the ICU. To identify factors that may predict extended stay in the ICU following surgery for ovarian cancer. **Methods:** A retrospective review was conducted of patients who underwent surgery for ovarian cancer and were admitted to the ICU at a single tertiary hospital center between January 2004 and December 2023. Patient demographics, clinical characteristics, and perioperative variables were analyzed. **Results:** Of the 74 patients included, 36.5% had an ICU stay of at least 48 h. Factors associated with prolonged ICU stay included higher Body Mass Index (BMI) and time of surgery duration. In contrast, a greater body temperature at the end of the operation was linked to a decrease in the length of ICU stay. **Conclusions:** These findings provide valuable insights for preoperative counseling and resource allocation, enhancing postoperative care for ovarian cancer patients. Additional research is required to confirm these data and investigate any other factors that may predict extended stay in the ICU.

## Simple Summary

Although ovarian cancer surgery frequently necessitates admission to an intensive care unit (ICU), not all patients require extended ICU stays. Our study aimed to identify what factors predict longer ICU stay after ovarian cancer surgery. We extracted information from 74 patients who received ovarian cancer surgery and were admitted to the ICU. We found that higher body mass index (BMI), longer operation duration, and greater blood loss increased the chance of prolonged ICU stays. Conversely, the use of epidural anesthesia and a higher body temperature at the end of surgery reduced the likelihood. Understanding these factors can help clinicians better manage resources and enhance patient recovery following ovarian cancer surgery.

## 1. Introduction

Ovarian cancer stands as the primary cause of mortality among women with any gynecological cancers [1]. The incidence of ovarian cancer displays significant variation worldwide, with higher rates reported in developed countries [2]. The likelihood of developing ovarian cancer increases with age, predominantly affecting postmenopausal women, highlighting age as a significant risk factor [3]. There are also other factors, like genetic, environmental, and lifestyle status, that may influence this risk [4]. Unfortunately, patients with ovarian cancer are frequently diagnosed at advanced stages, which significantly decreases survival rates [5,6]. The treatment process usually involves a combination of surgery and chemotherapy [7]. Surgical procedures aim for complete or optimal debulking (residual disease <1 cm), requiring bowel, urinary tract, or liver resection, splenectomy, peritonectomy, diaphragmatic stripping, or pelvic and paraaortic lymphadenectomy, in addition to the standard procedures of hysterectomy, adnexectomy, and omentectomy [8]. Given the disease characteristics, patient-related factors, and the complexity of surgical interventions, women undergoing debulking surgery for ovarian cancer are often transferred to the intensive care unit (ICU) postoperatively, with reported frequencies ranging from 20% to 48% in the literature [9,10].

Unplanned ICU admissions are associated with increased mortality, but in many institutions they may routinely admit patients to the ICU after certain ovarian cancer debulking procedures, including primary debulking or after hyperthermic intraperitoneal chemotherapy administration [11]. However, it is imperative to note that routine ICU admissions may be deemed unnecessary and contribute to increased morbidity [12]. Therefore, to identify the appropriate candidates for ICU admission, several studies have described different patient characteristics and perioperative factors associated with increased risk for ICU admission after surgery for gynecological malignancies [13]. Indeed, factors like preoperative nutritional status, advanced age, long operative time, and intestinal resection have been evaluated as predictors of length of ICU stay following surgery for ovarian cancer [12,14,15], with prolonged ICU stay often defined as a duration exceeding 48 h. These predictors can also serve as proxies for overall hospital length of stay and associated morbidity. Our study seeks to contribute to this ongoing research by delineating a patient profile that allows for predicting those patients who are likely to undergo a prolonged ICU stay after surgery for ovarian cancer.

## 2. Materials and Methods

A single center retrospective review of patients admitted to the ICU following primary, interval or late debulking surgery for ovarian cancer was conducted at a tertiary institution between January 2004 and December 2023. The types of surgeries performed on patients requiring ICU transfer include cytoreductive surgery, extensive peritoneal resections, and procedures involving major vascular or gastrointestinal interventions. ICU admissions were specifically due to significant perioperative complications, such as hemodynamic instability requiring vasopressor support, respiratory compromise necessitating advanced monitoring or intervention, or major surgical complications, including excessive intraoperative blood loss or the need for complex postoperative care. Ethical approval for this study was obtained from the hospital health ethics committee. All patients underwent surgery performed by two specialized gynecologic oncologists, following the guidelines of the European Society of Gynecological Oncology (ESGO), and always with the maximum effort to achieve no residual disease. The anaesthetic management was carried out by a standard and dedicated team of anesthesiologists with expertise in gynecologic oncology surgeries. The decision to transfer the patient to the ICU was made through a collective agreement among the attending surgeon, anesthesiologist, and intensive care unit medical team, based on the patient’s need for vital support, hemodynamic instability, and respiratory indications, guided by established clinical criteria to ensure consistency and minimize bias. These criteria include, but are not limited to, the following indicators of hemodynamic instability or critical condition: persistent hypotension or tachycardia, significant hypoxia, metabolic derangements (e.g., severe acidosis with or hyperkalemia), coagulopathy, oliguria or acute kidney injury (based on KDIGO criteria), and persistent elevated blood lactate levels. The duration of anesthesia is evaluated as a contributing factor, particularly for prolonged procedures (>4 h), which may increase the risk of complications such as fluid shifts, hypothermia, or prolonged ventilator dependence, thereby influencing the decision for ICU admission. To avoid multiple indication for ICU admission, each patient was assigned to a single primary category based on the dominant clinical condition, as determined through a consensus among the attending surgeon, anesthesiologist, and ICU medical team.

All data were obtained from the hospital’s computerized patient data system. Patients demographic and clinical characteristics such as age, BMI, preoperative albumin serum levels, American Society of Anesthesiologists (ASA) classification and intraoperative variables such as ascites, pleural effusion, blood loss, operation duration, bowel resection, intraoperative complications, epidural anesthesia and body temperature at the end of the operation were recorded. The primary outcome measure in the study was the length of ICU stay. The first group consisted of patients that were admitted to the ICU <48 h and the second group ≥48 h. The 48-h cut-off was selected because it has been previously used in similar studies to define prolonged ICU stay [12,15]. Furthermore, it was chosen as it represented the median length of stay among the patients in this study.

Continuous variables are presented with mean and standard deviation (SD). Group comparisons for continuous variables were made using the *t*-test for independent samples. For categorical variables group comparisons were made using the chi-squared test. Univariable and multivariable binary logistic regression analysis was performed to assess the independent predictors of prolonged ICU stay (0: <48 h, 1: ≥48 h). The multivariable binary logistic regression model included all predictor variables that exhibited a *p*-value less than 0.200 in the univariable binary logistic regression analysis. This threshold (0.200) was selected in order not to miss impactful predictors in the multivariable due to the rather small sample size. A lower threshold was considered as well (*p*-value less than 0.150).

To assess the validity and generalizability of the obtained results, a bootstrap validation procedure with 10,000 iterations was employed. In each iteration, a bootstrap sample was drawn with replacement from the original dataset, and the multivariable model was refitted on this resampled data including the same number of predictors as in the original analysis. The fitted model was then used to predict the outcome of the ICU stay (0: <48 h, 1: ≥48 h) on the original dataset, and the area under the receiver operating characteristic curve (AUROC) based on these predictive probabilities was computed to evaluate model performance. The distribution of the AUROC values across all iterations was summarized to provide an estimate of model performance and its variability. The receiver operating characteristic curve (ROC) of the original sample was visualized as well using the roc function (R package “pROC”).

For visualization purposes a forest plot was also employed to display the results of the univariable binary logistic regression using the R package “forestplot”. The level of statistical significance was set at a = 0.05. The analyses were conducted using the SPSS software (version 22.0) and the R programming language (version 4.4.1).

## 3. Results

A total of 74 out of 1045 patients who underwent surgery for ovarian cancer were admitted postoperatively to the ICU. Forty-seven patients (63.5%) had an ICU stay of less than 48-h (Group A) and 27 (36.5%) stayed at least 48-h (Group B) in the ICU (Table 1). Stage III (67.6%) and IV (29.7%) represented the majority of the cases, while only one case was stage I (1.4%) and one case was stage V (1.4%) (Table 1). Thirty-four patients (45.9%) required support due to hemodynamic instability, 39 patients (52.7%) required mechanical ventilation, and 1 patient (1.4%) required renal replacement therapy (Table 1).

Comparing group A and group B regarding both the preoperative and intraoperative variables revealed that the preoperative variables did not exhibit significant differences between the two groups, except for the number of neoadjuvant chemotherapy (NACT) cycles, which showed a significant difference among patients who underwent interval/late debulking surgery (Table 2).

On the other hand, among the intraoperative variables, the time of surgery exhibited significant differences between the two groups (Table 2) with a mean value of 312.13 (SD = 77.09) in group A and a statistically significantly larger mean of 363.15 (SD = 91.35) in group B (*p* = 0.013). In addition, the postsurgery body temperature obtained mean 35.87 (SD = 0.59) in group A and a statistically significantly smaller mean of 35.06 (SD = 0.94) in group B (*p* < 0.001). Of note, the intraoperative blood loss was marginally not statistically significantly different between the two groups (*p* = 0.162), exhibiting a mean of 757.96 (SD = 626.91) in group A and a much large mean value of 969.23 (SD = 561.26) in group B (Table 2).

**Figure 1:**
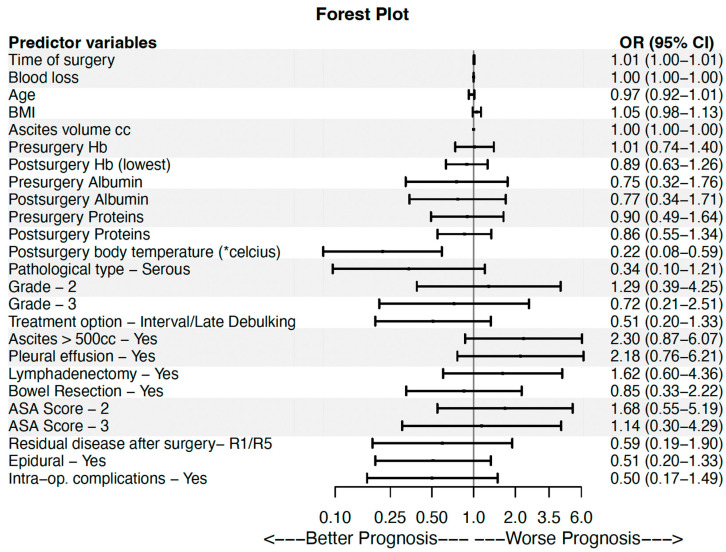
Univariable binary logistic regression results. The 2nd column displays the odds ratio estimation value (black square) along with the 95% confidence interval. The 3rd column displays their specific values. OR stands for odds ratio and OR 95% CI for the 95% confidence interval of the odds ratio. The axis labels “Worse prognosis” (>1) and “Better prognosis” (<1) indicate whether a predictor is favoring or not long ICU stay, respectively.

Similar results were obtained in the case of the univariable binary logistic regression analysis (Table 3). In particular, the time of surgery exhibited a statistically significant odds ratio (OR) of 1.007 (95% Confidence Interval (CI): 1.001–1.013, *p* = 0.016), namely, the odds of ICU stay ≥48 h were 1.007 times greater compared to ICU stay <48 h, for each increase of the surgery time by one. In other words, as the time of surgery increased, the patient was more likely to belong in group B. Τhe postsurgery body temperature obtained an OR of 0.220 (95% CI: 0.082–0.589, *p* = 0.003), namely as the postsurgery body temperature increased, the patient was more likely to belong in group A. The results of this analysis are visually displayed as well in Figure 1. All variables with a *p*-value less than 0.200 in the univariable binary logistic regression were included in the multivariable model (Table 3). More specifically, ten variables were included in the multivariable analysis. Of these, three factors exhibited statistical significance, the time of surgery (OR: 1.017, 95% CI: 1.002–1.033, *p* = 0.025), and the BMI (OR: 1.215, 95% CI: 1.021–1.446, *p* = 0.028), were associated with increased risk for admission to the ICU for ≥48 h, while higher postsurgery body temperature (OR: 0.082, 95% CI: 0.013–0.507, *p* = 0.007) significantly decreased the likelihood of prolonged ICU stay (Table 3).

When considering a lower threshold (0.150) regarding the *p*-value in the univariable binary logistic regression for the respective variables to be included in the multivariable model, seven (instead of ten) variables were included in the multivariable model since Blood loss, Treatment option and Epidural did not satisfy the entry criterion (*p*-value < 0.150). It was observed that the results did not change in terms of statistical significance. In particular, the same three factors remained statistically significanct in the multivariable analysis, namely, the time of surgery (OR: 1.017, 95% CI: 1.003–1.032, *p* = 0.021), the BMI (OR: 1.223, 95% CI: 1.035–1.447, *p* = 0.018), and postsurgery body temperature (OR: 0.087, 95% CI: 0.016–0.460, *p* = 0.004). All other factors did not exhibit a statistically significant impact on ICU stay.

The validity and generalizability of the obtained results was then assessed with the proposed bootstrap-based validation procedure. The distribution of the AUROC values across all iterations was summarized in the case that the *p*-value threshold was set to 0.200 to provide an estimate of model performance and its variability. It was found that the median AUROC across the 10,000 iterations was 0.859, the mean was 0.852, the 1st quartile value was 0.827 and the 3rd quartile value was 0.884. The standard deviation of the AUROC was found to be 0.044. The 95% confidence interval of the AUROC was 0.746–0.915. It should be noted that the calculation of the AUROC using the predictive probabilities that were obtained based on the fitted model deriving from the original dataset (instead of the bootstrap samples in the above procedure) led to a value of 0.926.

## 4. Discussion

Prolonged staying in the intensive care unit (ICU) after ovarian cancer surgery constitutes a critical element in delivering optimal patient care. The identification of factors influencing prolonged ICU stay enables healthcare providers to judiciously allocate resources and formulate strategies for minimizing complications, thereby enhancing recovery of patients after debulking surgery for ovarian cancer. Effectively managing and mitigating these factors in routine clinical practice is imperative to streamline patient care and diminish extended ICU utilization after ovarian cancer surgery. The implementation of enhanced perioperative care protocols (Enhanced Recovery After Surgery—ERAS) and attentive monitoring systems empowers healthcare providers to proactively identify individuals at increased risk for prolonged ICU stays. However, this potential impact requires further study to better understand its role and effectiveness in different clinical settings.

In our study, the most common indications for ICU admission were mechanical ventilation support after major surgery and hemodynamic instability. Extensive traumatic surgical procedures aimed at achieving optimal debulking result in higher rates of significant blood loss, intraoperative complications, and anesthesia-related challenges, highlighting the need for meticulous fluid management and mechanical respiratory support. Approximately 21–36% of these patients require postoperative ICU admission [15]. In addition to patients’ age, comorbidities, and nutritional status, which play a pivotal role in determining ICU admission, the type and duration of surgery also contribute significantly to the decision-making process [13]. While some advancements in perioperative care occurred during this period, our institutional practice patterns remained relatively consistent. Regarding Interval Debulking Surgery (IDS), its utilization did increase following the publication of studies demonstrating its non-inferiority to Primary Debulking Surgery (PDS). However, IDS was performed even in the early years of our study. This ensures that variations in IDS and PDS utilization do not introduce bias in surgical techniques. Our study includes both IDS and PDS patients, which helps minimize the impact of evolving neoadjuvant chemotherapy (NACT) practices on ICU admission trends. Furthermore, in our statistical analysis, although NACT was statistically significant between the two groups, this significance was lost in both univariable and multivariable analyses (Table 2 and Table 3). These data are also confirmed by a recent systematic review, which found that NACT is not a strong prognostic factor for ICU admission [13]. Similarly, no statistical significance was observed between patients who underwent extensive bowel resections or lymphadenectomy in either univariable or multivariable analyses. Additionally, no significant correlation was found with intraoperative complications.

Age has been identified as an independent predictor for prolonged ICU stay after surgery for gynecologic cancer according to several studies [12,14,16,17]. In our study age was not significantly different between the groups of patients. Malnutrition is prevalent among oncologic patients and is correlated with a variety of postoperative complications [12,14]. Serum albumin levels above 3.5 g/dL, as a proxy for nutritional status, have been associated with a reduced risk of prolonged ICU stay [12]. However, in our study there was no significant difference in preoperative albumin levels between the two groups. While low preoperative albumin levels are often associated with poorer surgical outcomes, our cohort may have benefited from preoperative nutritional optimization strategies, such as dietary supplementation or parenteral nutrition in select cases, which could have attenuated the impact of hypoalbuminemia on postoperative recovery. Furthermore, the expertise of our consistent surgical and anesthesiology team, specialized in gynecologic oncology, likely contributed to optimized intraoperative management, potentially reducing the influence of preoperative albumin levels on ICU requirements. The non-significant association may also be influenced by the variability in albumin levels within our cohort, which, as reported, showed a relatively narrow range, potentially limiting the ability to detect a statistically significant effect Among other patient characteristics, BMI was associated with increased risk for ICU admission for ≥48 h. Obesity has been associated with an increased risk of postoperative complications after ovarian cancer surgery, particularly wound complications, infections and coagulopathy [18,19]. From literature review, Wolfberg et al. [20] found a correlation between a BMI greater than 30 and an elevated rate of ICU admission, while Yao et al. [9] linked BMI with ICU admission within 30 days postoperatively. In our study, we observed that for each increase in BMI grade, the odds of prolonged ICU stay ≥48 h increased by 21.5%. Different scoring systems have been developed to classify patients based on comorbidities and performance status, aiming to identify those who may require more intensive postoperative care [21]. In our study, we utilized the American Society of Anesthesiologists physical status (ASA) classification [22], to assess the medical comorbidities and estimate likelihood of disease, but we did not observe any significant difference between the two groups. In the last few years, frailty has emerged as a predictor of adverse outcomes following ovarian cancer surgery [23]. Frailty is a condition in which the individual is in a vulnerable state at increased risk of adverse health outcomes and/or dying when exposed to a stressor [24]. Yao et al. demonstrated that frailty remained an independent predictor of 30-day ICU admission postoperatively [9]. Frailty is common among patients with advanced epithelial ovarian cancer and correlated with increased rates of perioperative morbidity and mortality. Although it is age-related, it does not necessarily follow a linear progression with increasing age [23]. 

Regarding intraoperative outcomes, prolonged surgical procedures were found to increase the likelihood of extended ICU admissions. A longer duration of surgery often indicates more complex procedures, a higher risk of intraoperative complications, and the necessity for administering larger and more prolonged doses of anesthetic agents. This makes the recovery process more challenging for these patients and significantly increases the likelihood of their transfer to the ICU. Indeed, the estimated duration of surgery differed significantly between the two groups in our study. Also the lack of a significant association between preoperative ascites and ICU stay duration in our study may be attributed to effective intraoperative management. Although ascites is a marker of advanced disease in gynecologic oncology patients, it may not directly correlate with postoperative complications due to complete intraoperative drainage of ascites, which may mitigate its impact on recovery.

Furthermore, epidural anesthesia has been shown to improve survival in ovarian cancer patients, likely due to its ability to reduce the stress response [25,26]. In our study, we observed that epidural anesthesia was associated with a reduction in ICU stay, although this finding was not statistically significant. The anesthetic protocols applied in our study, including epidural administration of Ropivacaine^®^ 0.2–0.3% without opioids, followed standardized institutional guidelines that have remained unchanged since the study’s initiation This effect may be attributed to decreased requirements for sedative drugs and enhanced pain management, which could facilitate more efficient patient resuscitation after surgery and promote a smoother recovery from anesthesia.

Hypothermia has been associated with early postoperative complications and mortality among women underwent cytoreductive surgery for ovarian cancer [27]. Visceral exposure and anesthesia-induced dysfunction of thermoregulatory control are primary factors contributing to intraoperative hypothermia [28]. Neuraxial anesthesia prevents vaso-constriction and shivering in blocked areas. Therefore, unwarmed patients become hypothermic. Hypothermia has been shown to have detrimental effects on various physiological processes, including coagulation, susceptibility to infections, drug metabolism and vasoconstriction [29]. Kaufner et al. demonstrated that prewarming patients undergoing ovarian cancer cytoreductive surgery to 43 °C using a forced air warming gown connected to a forced air warmer, during epidural catheter placement and induction of anesthesia, correlated with reduced drop in core temperature and the maintenance of normothermia throughout the surgery [30]. On the contrary, Long et al. [31] showed that although hypothermia is common among such patients, it was not correlated with increased postoperative complications. In our study, the use of intraoperative forced-air warming and the administration of pre-warmed intraoperative fluids contributed to maintaining patient normothermia. Postoperative core temperature, measured via a bladder catheter immediately upon completion of surgery and prior to ICU transfer, was inversely associated with the risk of prolonged ICU admission (≥48 h), with higher core temperatures correlated with shorter ICU stays.

The robustness of this investigation is grounded in the execution of all primary procedures within a certified gynecological oncological center with accreditation for advanced ovarian cancer surgery and by two proficient gynecological oncology surgeons. Additionally, the anesthetic interventions were administered by three experienced anesthesiologists well-versed in radical cytoreductive surgeries. Also, the ICU is a referral center for the broader region’s critical cases. However, certain constraints merit attention in our study. Notably, the retrospective, single-center design, the limited sample size, and the infrequent occurrence of events may have limited the ability to identify additional predictors. The primary reason for this discrepancy is the availability of monitoring equipment in the wards for gynecological oncological patients at our institution. Specifically, postoperative patients, even those requiring heightened observation the first 24 to 48 h, are managed directly in their ward beds using continuous monitoring systems, which reduces the need for ICU admission only in critical patients. This setup allows for close surveillance and timely intervention without transferring patients to the ICU, except in cases of critical instability where the decision is made postoperatively. Moreover, frailty status could only be assessed using the ASA score, as no other indices were consistently available. Intraoperative fluid balance was not analyzed due to insufficient documentation in the patient records. Additionally, Enhanced Recovery After Surgery (ERAS) protocols were officially implemented in our department after the majority of the study period, and thus did not influence patients in our study. These factors may be strong predictors of prolonged ICU stay, and their exclusion represents a limitation of the study, potentially impacting the generalizability of our findings. Considering the multivariable binary logistic regression model, an events-per-variable (EPV) calculation has been performed resulting in 2.70 and 3.86 corresponding to the different values of entering *p*-value threshold of 0.200 and 0.150, respectively. These values are rather low; however, the assessment of the validity and generalizability of the obtained results were also evaluated with the proposed bootstrap-based procedure showing satisfactory robustness. For these reasons, there is a need for more extensive and larger studies involving multiple gynecologic oncology centers. This perspective could support as well the development of a risk score for the ICU unit. Our findings on predictors of prolonged ICU stay after ovarian cancer surgery should also be viewed in light of existing evidence on surgical approach and patient-related factors affecting postoperative recovery. Previous systematic reviews comparing laparoscopy and laparotomy in advanced ovarian cancer have highlighted differences in perioperative outcomes and recovery trajectories, which may indirectly influence postoperative ICU needs [32]. Additionally, literature on managing elderly and frail ovarian cancer patients emphasizes how advanced age and frailty are important factors in perioperative morbidity and extended ICU use [33]. Including these perspectives could further enhance the discussion on how preoperative and intraoperative factors fit into comprehensive risk stratification models.

## 5. Conclusions

Specific perioperative criteria can predict whether a patient with ovarian cancer may require a prolonged ICU stay. These findings facilitate appropriate preoperative counseling and empower gynecologic-oncologists to identify these patients, that will benefit the most from postoperative ICU admission, contributing to improved resource management in favor of patients.

The data analysis of this retrospective study revealed that, in addition to factors contributing to extended ICU utilization, such as increased BMI and prolonged surgery duration, an additional factor was identified that could reduce this likelihood: a higher body temperature at the end of the operation.

## Figures and Tables

**Table 1:** Patient characteristics.

Patient characteristics	*n* = 74 (%)	<48 h *n* = 47 (63.5%)	≥48 h *n* = 27 (36.5%)
Stage, *n* (%)			
I	1 (1.4%)	1 (2.1%)	0 (0.0%)
III	50 (67.6%)	30 (63.8%)	20 (74.1%)
IV	22 (29.7%)	16 (34.0%)	6 (22.2%)
V	1 (1.4%)	0 (0.0%)	1 (3.7%)
Tumor grade, *n* (%)			
Grade 1	19 (25.7%)	12 (25.5%)	7 (25.9%)
Grade 2	28 (37.8%)	16 (34.0%)	12 (44.4%)
Grade 3	27 (36.5%)	19 (40.4%)	8 (29.6%)
Cause of icu admission			
Hemodynamic support	34 (45.9%)	25 (53.2%)	9 (33.3%)
Mechanical Ventilation	39 (52.7%)	22 (46.8%)	17 (63.0%)
Renal replacement therapy	1 (1.4%)	0 (0.0%)	1 (3.7%)

**Table 2:** Comparison of the perioperative variables between ICU < 48 h and ICU ≥ 48 h.

Perioperative/Intraoperative Variables	<48 h *n* = 47 (63.5%)	≥48 h *n* = 27 (36.5%)	Mean Difference (95% CI)	*p*-Value
Preoperative				
Age (years)	60.79 (10.77) 60.00 (IQR: 14)	56.82 (11.02) 58.00 (IQR: 15)	3.97 (−1.26, 9.20)	0.134
ΒΜΙ	29.29 (6.88) 28.30 (IQR: 13)	31.77 (6.91) 33.70 (IQR: 13)	−2.48 (−5.80, 0.84)	0.140
Ascites > 500cc				0.091
No	27 (73.0%)	10 (27.0%)	-	
Yes	20 (54.1%)	17 (45.9%)	-	
Presurgery Hb (g/dL)	11.93 (1.43) 12.00 (IQR: 2)	11.97 (1.59) 12.00 (IQR: 2.3)	−0.04 (−0.75, 0.68)	0.928
Presurgery Albumin (g/dL)	3.93 (0.57) 3.90 (IQR: 0.7)	3.84 (0.63) 4.00 (IQR: 0.5)	0.09 (−0.20, 0.39)	0.517
Presurgery Proteins (g/dL)	6.81 (0.64) 6.90 (IQR: 0.8)	6.74 (1.08) 6.94 (IQR: 0.9)	0.07 (−0.34, 0.48)	0.734
Number of Chemotherapies	4.67 (1.13) 4.50 (IQR: 2)	3.73 (0.91) 4.00 (IQR: 1)	0.939 (0.15, 1.73)	**0.021**
Pathological type				0.086
Non-serous	5 (41.7%)	7 (58.3%)	-	
Serous	42 (67.7%)	20 (32.3%)	-	
Grade				0.595
1	12 (63.2%)	7 (36.8%)	-	
2	16 (57.1%)	12 (42.9%)	-	
3	19 (70.4%)	8 (29.6%)	-	
ASA Score				0.624
1	16 (69.6%)	7 (30.4%)	-	
2	19 (57.6%)	14 (42.4%)	-	
3	12 (66.7%)	6 (33.3%)	-	
Intraoperative				
Time of surgery (min)	312.13 (77.09) 300.00 (IQR: 120)	363.15 (91.35) 400.00 (IQR: 150)	−51.02 (−90.75, −11.29)	**0.013**
Blood loss (cc)	757.96 (626.91) 500.00 (IQR: 600)	969.23 (561.26) 750.00 (IQR: 550)	−211.27 (−509.22, 86.67)	0.162
Ascites volume (cc)	2368.42 (1498.17) 2000.00 (IQR: 1000)	2558.82 (1796.20) 2000.00 (IQR: 2750)	−190.40 (−1306.58, 925.77)	0.731
Treatment option				0.166
Primary Debulking	20 (55.6%)	16 (44.4%)	-	
Interval/Late Debulking	27 (71.1%)	11 (28.9%)	-	
Pleural effusion				0.142
No	37 (68.5%)	17 (31.5%)	-	
Yes	10 (50.0%)	10 (50.0%)	-	
Lymphadenectomy				0.338
No	33 (67.3%)	16 (32.7%)	-	
Yes	14 (56.0%)	11 (44.0%)	-	
Bowel Resection				0.742
No	26 (61.9%)	16 (38.1%)	-	
Yes	21 (65.6%)	11 (34.4%)	-	
Residual disease after surgery				0.378
R0	34 (60.7%)	22 (39.3%)	-	
R1/R5	13 (72.2%)	5 (27.8%)	-	
Epidural				0.166
No	20 (55.6%)	16 (44.4%)	-	
Yes	27 (71.1%)	11 (28.9%)	-	
Intra-op. complications				0.212
No	30 (58.8%)	21 (41.2%)	-	
Yes	17 (73.9%)	6 (26.1%)	-	
Postoperative				
Postsurgery Hb (lowest) (g/dL)	8.64 (1.13) 8.60 (IQR: 1.5)	8.42 (1.81) 7.80 (IQR: 1.8)	0.22 (−0.56, 1.00)	0.572
Postsurgery Albumin (g/dL)	2.16 (0.62) 2.20 (IQR: 1)	2.06 (0.63) 2.27 (IQR: 0.9)	0.10 (−0.21, 0.42)	0.522
Postsurgery Proteins (g/dL)	3.77 (1.14) 3.86 (IQR: 1.4)	3.58 (1.16) 3.60 (IQR: 1.3)	0.19 (−0.39, 0.77)	0.511
Postsurgery body temperature (*Celcius)	35.87 (0.59) 35.80 (IQR: 1)	35.06 (0.94) 34.95 (IQR: 1.2)	0.81 (0.38, 1.23)	**<0.001**
Cause of icu admission				0.131
Hemodynamic support	25 (73.5%)	9 (26.5%)	-	
Mechanical Ventilation	22 (56.4%)	17 (43.6%)	-	
Renal replacement therapy	0 (0.0%)	1 (100.0%)	-	
Perioperative				
Blood Transfusion	0.89 (1.71) 0.00 (IQR: 1)	0.44 (1.01) 0.00 (IQR: 0)	0.45 (−0.27, 1.17)	0.218

**Table 3:** Binary logistic regression results (univariable and multivariable). OR stands for Odds Ratio and OR 95% CI for the 95% Confidence Interval of the Odds Ratio. For categorical variables the reference category is displayed in parenthesis. For binary variables with values Yes/No, No is always the reference category.

	Univariable			Multivariable		
Predictor Variables	OR	OR 95% CI	*p*-Value	OR	OR 95% CI	*p*-Value
Time of surgery (min)	1.007	(1.001–1.013)	**0.016**	1.017	(1.002–1.033)	**0.025**
Blood loss (cc)	1.001	(1.000–1.001)	0.171	1.000	(0.998–1.002)	0.893
Age (years)	0.966	(0.923–1.011)	0.138	0.946	(0.849–1.054)	0.311
ΒΜΙ	1.054	(0.983–1.131)	0.140	1.215	(1.021–1.446)	**0.028**
Ascites volume (cc)	1.000	(1.000–1.000)	0.722			
Presurgery Hb (g/dL)	1.015	(0.736–1.401)	0.927			
Postsurgery Hb (lowest) (g/dL)	0.892	(0.632–1.260)	0.517			
Presurgery Albumin (g/dL)	0.754	(0.323–1.758)	0.513			
Postsurgery Albumin (g/dL)	0.767	(0.344–1.708)	0.516			
Presurgery Proteins (g/dL)	0.899	(0.492–1.643)	0.730			
Postsurgery Proteins (g/dL)	0.859	(0.548–1.344)	0.505			
Postsurgery body temperature (*celcius)	0.220	(0.082–0.589)	**0.003**	0.082	(0.013–0.507)	**0.007**
Pathological type—Serous (ref. Other types)	0.340	(0.096–1.205)	0.095	0.459	(0.060–3.531)	0.454
Grade			0.597			
2 (ref. 1)	1.286	(0.389–4.249)	0.680			
3 (ref. 1)	0.722	(0.208–2.508)	0.608			
Treatment option—Interval/Late Debulking (ref. Primary Debulking)	0.509	(0.195–1.331)	0.169	0.293	(0.033–2.629)	0.273
Ascites > 500cc—Yes	2.295	(0.868–6.065)	0.094	2.481	(0.234–26.327)	0.451
Pleural effusion—Yes	2.176	(0.763–6.207)	0.146	2.350	(0.146–37.863)	0.547
Lymphadenectomy—Yes	1.621	(0.602–4.361)	0.339			
Bowel Resection—Yes	0.851	(0.326–2.221)	0.742			
ASA Score			0.626			
2 (ref. 1)	1.684	(0.547–5.187)	0.364			
3 (ref. 1)	1.143	(0.305–4.289)	0.843			
Residual disease after surgery- R1/R5 (ref. R0)	0.594	(0.186–1.901)	0.380			
Epidural—Yes	0.509	(0.195–1.331)	0.169	1. 064	(0.112–10.079)	0.957
Intra-op. complications—Yes	0.500	(0.170–1.490)	0.216			

## Data Availability

We confirm that the data supporting the findings of this study are available upon request. However, due to privacy concerns and in accordance with ethical standards and regulations, the data will be provided in a deidentified format to ensure patient anonymity. Requests for data should be directed to the corresponding author via email at theodoulidisvasilis@yahoo.gr.

## Abbreviations

The following abbreviations are used in this manuscript:

ICU	Intensive Care Unit
BMI	Body Mass Index
ESGO	European Society of Gynecological Oncology
SD	Standard Deviation
ASA	American Society of Anesthesiologists
NACT	NeoAdjuvant ChemoTherapy
OR	Odds Ratio
CI	Confidence Interval
0	Enhanced Recovery After Surgery
KDIGO	Kidney Disease: Improving Global Outcomes

## References

[1] PDQ Adult Treatment Editorial Board. PDQ Cancer Information Summaries [Internet]. National Cancer Institute (US); Bethesda (MD): Ovarian Epithelial, Fallopian Tube, and Primary Peritoneal Cancer Treatment (PDQ^®^): Health Professional Version. https://www.ncbi.nlm.nih.gov/books/NBK66007/.

[2] Cabasag C.J., Fagan P.J., Ferlay J., Vignat J., Laversanne M., Liu L., van der Aa M.A., Bray F., Soerjomataram I. (2022). Ovarian cancer today and tomorrow: A global assessment by world region and Human Development Index using GLOBOCAN 2020. Int. J. Cancer.

[3] Chan J.K., Urban R., Cheung M.K., Osann K., Husain A., Teng N.N., Kapp D.S., Berek J.S., Leiserowitz G.S. (2006). Ovarian cancer in younger vs older women: A population-based analysis. Br. J. Cancer.

[4] Momenimovahed Z., Tiznobaik A., Taheri S., Salehiniya H. (2019). Ovarian cancer in the world: Epidemiology and risk factors. Int. J. Women’s Health.

[5] SEER Cancer Statistics Review, 1975–2001. http://www.seer.cancer.gov/csr/1975_2001/.

[6] Arora T., Mullangi S., Lekkala M.R. Ovarian Cancer. [Updated 2023 Jun 18]. In: StatPearls [Internet]. Treasure Island (FL): StatPearls Publishing; 2023 Jan-. https://www.ncbi.nlm.nih.gov/books/NBK567760/.

[7] Coleridge S.L., Bryant A., Kehoe S., Morrison J. (2021). Neoadjuvant chemotherapy before surgery versus surgery followed by chemotherapy for initial treatment in advanced ovarian epithelial cancer. Cochrane Database Syst. Rev..

[8] Querleu D., Planchamp F., Chiva L., Fotopoulou C., Barton D., Cibula D., Aletti G., Carinelli S., Creutzberg C., Davidson B. (2017). European Society of Gynaecological Oncology (ESGO) Guidelines for Ovarian Cancer Surgery. Int. J. Gynecol. Cancer.

[9] Yao T., DeJong S.R., McGree M.E., Weaver A.L., Cliby W.A., Kumar A. (2019). Frailty in ovarian cancer identified the need for increased postoperative care requirements following cytoreductive surgery. Gynecol. Oncol..

[10] Davidovic-Grigoraki M., Thomakos N., Haidopoulos D., Vlahos G., Rodolakis A. (2016). Do critical care units play a role in the management of gynaecological oncology patients? The contribution of gynaecologic oncologist in running critical care units. Eur. J. Cancer Care.

[11] Ross M.S., Burriss M.E., Winger D.G., Edwards R.P., Courtney-Brooks M., Boisen M.M. (2018). Unplanned postoperative intensive care unit admission for ovarian cancer cytoreduction is associated with significant decrease in overall survival. Gynecol. Oncol..

[12] Díaz-Montes T.P., Zahurak M.L., Bristow R.E. (2007). Predictors of extended intensive care unit resource utilization following surgery for ovarian cancer. Gynecol. Oncol..

[13] Hamaguchi R., Ito T., Narui R., Morikawa H., Uemoto S., Wada H. (2020). Postoperative Admission in Critical Care Units Following Gynecologic Oncology Surgery: Outcomes Based on a Systematic Review and Authors’ Recommendations. Vivo.

[14] Amir M., Shabot M., Karlan B.Y. (1997). Surgical intensive care unit care after ovarian cancer surgery: An analysis of indications. Am. J. Obstet. Gynecol..

[15] Collins A., Spooner S., Horne J., Chainrai M., Runau F., Bourne T., Moss E.L., Davies Q., Chattopadhyay S., Bharathan R. (2021). Peri-operative Variables Associated with Prolonged Intensive Care Stay Following Cytoreductive Surgery for Ovarian Cancer. Anticancer. Res..

[16] Brooks S.E., Ahn J., Mullins C.D., Baquet C.R. (2002). Resources and use of the intensive care unit in patients who undergo surgery for ovarian carcinoma. Cancer.

[17] Ruskin R., Urban R.R., Sherman A.E., Chen L.-L., Powell C.B., Burkhardt D.H. (2011). Predictors of Intensive Care Unit Utilization in Gynecologic Oncology Surgery. Int. J. Gynecol. Cancer.

[18] Cai B., Li K., Li G. (2022). Impact of Obesity on Major Surgical Outcomes in Ovarian Cancer: A Meta-Analysis. Front. Oncol..

[19] Smits A., Lopes A., Das N., Kumar A., Cliby W., Smits E., Bekkers R., Massuger L., Galaal K. (2015). Surgical morbidity and clinical outcomes in ovarian cancer—the role of obesity. BJOG Int. J. Obstet. Gynaecol..

[20] Wolfberg A.J., Montz F.J., E Bristow R. (2004). Role of obesity in the surgical management of advanced-stage ovarian cancer. J. Reprod. Med..

[21] Sobol J.B., Wunsch H. (2011). Triage of high-risk surgical patients for intensive care. Crit. Care.

[22] Pedrosa E., Silva M., Lobo A., Barbosa J., Mourao J. (2021). Is the ASA Classification Universal?. Turk. J. Anaesthesiol. Reanim..

[23] Kumar A., Langstraat C.L., DeJong S.R., McGree M.E., Bakkum-Gamez J.N., Weaver A.L., LeBrasseur N.K., Cliby W.A. (2017). Functional not chronologic age: Frailty index predicts outcomes in advanced ovarian cancer. Gynecol. Oncol..

[24] Walston J., Hadley E.C., Ferrucci L., Guralnik J.M., Newman A.B., Studenski S.A., Ershler W.B., Harris T., Fried L.P. (2006). Research Agenda for Frailty in Older Adults: Toward a Better Understanding of Physiology and Etiology: Summary from the American Geriatrics Society/National Institute on Aging Research Conference on Frailty in Older Adults. J. Am. Geriatr. Soc..

[25] Shen H., Pang Q., Gao Y., Liu H. (2023). Effects of epidural anesthesia on the prognosis of ovarian cancer—a systematic review and meta-analysis. BMC Anesthesiol..

[26] Tseng J.H., Cowan R.A., Afonso A.M., Zhou Q., Iasonos A., Ali N., Thompson E., Sonoda Y., O’CEarbhaill R.E., Chi D.S. (2018). Perioperative epidural use and survival outcomes in patients undergoing primary debulking surgery for advanced ovarian cancer. Gynecol. Oncol..

[27] Moslemi-Kebria M., El-Nashar S.A.M., Aletti G.D., Cliby W.A. (2012). Intraoperative Hypothermia During Cytoreductive Surgery for Ovarian Cancer and Perioperative Morbidity. Obstet. Gynecol..

[28] Xiong J., Kurz A., Sessler D.I., Plattner O., Christensen R., Dechert M., Ikeda T. (1996). Isoflurane Produces Marked and Nonlinear Decreases in the Vasoconstriction and Shivering Thresholds. Anesthesiology.

[29] Sessler D.I. (2016). Perioperative thermoregulation and heat balance. Lancet.

[30] Kaufner L., Niggemann P., Baum T., Casu S., Sehouli J., Bietenbeck A., Boschmann M., Spies C.D., Henkelmann A., von Heymann C. (2019). Impact of brief prewarming on anesthesia-related core-temperature drop, hemodynamics, microperfusion and postoperative ventilation in cytoreductive surgery of ovarian cancer: A randomized trial. BMC Anesthesiol..

[31] Long K.C., Tanner E.J., Frey M., Leitao M.M., Levine D.A., Gardner G.J., Sonoda Y., Abu-Rustum N.R., Barakat R.R., Chi D.S. (2013). Intraoperative hypothermia during primary surgical cytoreduction for advanced ovarian cancer: Risk factors and associations with postoperative morbidity. Gynecol. Oncol..

[32] Alletti S.G., Capozzi V.A., Rosati A., De Blasis I., Cianci S., Vizzielli G., Uccella S., Gallotta V., Fanfani F., Fagotti A. (2019). Laparoscopy vs. laparotomy for advanced ovarian cancer: A systematic review of the literature. Minerva Medica.

[33] Tortorella L., Vizzielli G., Fusco D., Cho W.C., Bernabei R., Scambia G., Colloca G. (2017). Ovarian Cancer Management in the Oldest Old: Improving Outcomes and Tailoring Treatments. Aging Dis..

